# Increasing CHO cell harvest efficiency with cyclical cake filtration

**DOI:** 10.1007/s00253-026-14021-7

**Published:** 2026-10-01

**Authors:** Chloe Jerrold-Jones Pynos, Tizian Bucher, Andry Mannone, Abiram Gopalakrishnan, Jan Ott

**Affiliations:** 1 DrM, Inc., 2125 Center Ave. Suite 507, Fort Lee, NJ 07024 USA; 2 DrM Dr. Müller AG, Alte Landstrasse 415, 8708 Männedorf, Switzerland; 3 ZHAW Zurich University of Applied Sciences, Institute of Chemistry and Biotechnology, Centre for Cell Cultivation Techniques, Tissue Engineering, and Medical Biology, Grüentalstrasse 14, 8820 Wädenswil, Switzerland

**Keywords:** CHO cell harvest, Clarification, Filtration, Body feed filtration, Cake filtration, Alluvial filtration, Depth filtration, Impurity removal, Scale-up, Processing efficiency

## Abstract

**Abstract:**

Efficient clarification of bioreactor harvests remains a critical bottleneck in biomanufacturing, directly impacting throughput, product yield, and operational costs. Current industry practice typically relies on depth filtration due to its effective removal of particulates and impurities; however, depth filters are prone to clogging, require oversized filter areas, and can reduce product yield through non-specific adsorption. Alluvial filtration addresses some of these limitations by using a diatomaceous earth (DE) filter cake to protect the underlying media and extend filtration capacity. Cyclical cake filtration further leverages the advantages of diatomaceous earth (DE) cake filtration through multi-cycle cloth regeneration. This study examines cyclical cake filtration and compares it with traditional and alluvial depth filtration regarding performance and scalability. Cyclical cake filtration maintained higher flow rates than depth filtration and exhibited no clogging under the tested conditions. With the addition of DE and various ion-exchange resins, cyclical cake filtration achieved filtrate turbidities as low as 2.8 NTU while maintaining product yields of 91–94%. DNA and host cell protein concentrations were reduced by up to 95% and 62%, respectively, approaching levels achieved by conventional two-stage clarification processes. Scale-up studies demonstrated comparable flow rate behavior and filtrate quality across three filtration scales spanning an 18-fold increase in filter area. Product yield remained consistent across scales, while impurity levels and turbidity exhibited minor variation. A commercial scale assessment for processing 2000 L of Chinese hamster ovary (CHO) culture estimated a primary clarification filter area requirement of 0.88 m^2^ for cyclical cake filtration, compared with 16.8 m^2^ and 28.5 m^2^ for alluvial and depth filtration, respectively. When processing time and filter area were combined into a proposed efficiency parameter, cyclical cake filtration demonstrated the most favorable performance. These results demonstrate that cyclical cake filtration is a scalable and efficient clarification technology capable of reducing process risk and consumable requirements while maintaining high product recovery and consistent filtration performance, and offers options for competitive impurity removal.

**Key points:**

• *Cyclical cake filtration offers an alternative to depth and alluvial filtration.*

• *More efficient cell harvest if combined filter area and processing time are considered.*

• *Lower process risk, filter area requirement, waste, and cost.*

## Introduction

With advancements in upstream design and the growing demand for pharmaceuticals and therapies, biomanufacturers are prioritizing efficient production processes that maximize product yield and recovery (Auerbach 2025; Sharma et al. 2017; Thakur et al. 2020). After a culture is grown in a bioreactor, clarification functions as the first step of removing cell debris, particulates, and ideally some impurities from the upstream harvest. Clarification, sometimes referred to as “midstream,” serves as the gateway into downstream processing. Because downstream operations are known to be some of the most cost-intensive steps of bioproduction (Sharma et al. 2017; Rooij et al. 2019), the quality of clarified feed entering this phase is a critical determinant of overall process efficiency and manufacturing costs. Clarification is often a multi-stage operation, where primary clarification removes larger cell debris and secondary clarification removes finer impurities and particulates.

**Fig. 1:**
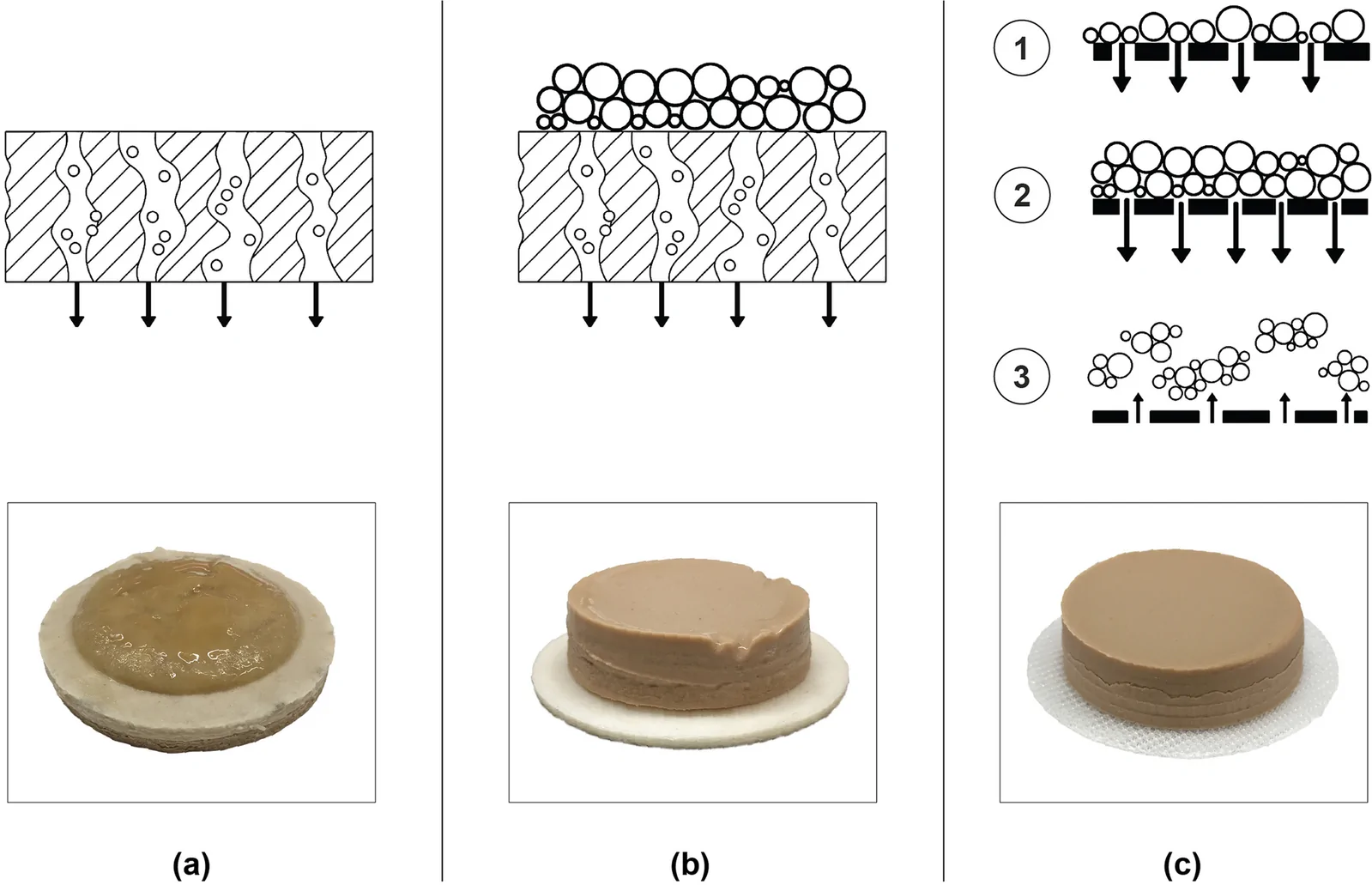
Illustrations of how filters behave and actual photos of filter cloths and cake for **a** depth filters, **b** alluvial filters, and **c** cyclical cake filters

For primary clarification, centrifuges and depth filters are popular options. For secondary clarification, typically another depth filter is used (Sharma et al. 2017). According to the 16th Annual Report and Survey of Biopharmaceutical Manufacturing Capacity and Production (2019), 79% of respondents reported using depth filtration. Despite their widespread adoption in the industry, depth filters present several limitations, particularly with commercial scale manufacturing of 2000 L or greater. Upstream variability or high cell densities (e.g., > 25 × 10^6^ cells mL^−1^) can lead to dramatic decrease of flow rates and early clogging of depth filters (Minow et al. 2014). Cells commonly are captured directly within the tortuous path of the thick depth filter media (Fig. 1a), forming a gel-like layer on the top. Non-uniform flow and pressure increases may trigger cells to break free and cause turbidity breakthroughs, jeopardizing reliable clarity requirements for filtrate (Nejatishahidein et al. 2022). Furthermore, once a depth filter medium is clogged, the cartridge is considered spent and must be replaced. To ensure a complete batch can be processed without risk of early clogging, various sources in the literature suggest purposefully oversizing the filter area, referencing safety factors between 1.2 and 2.3 (Laska et al. 2005; Thakur et al. 2020; Dryden et al. 2021). Compensating this way may solve the problem of unreliable results when scaling up, but it comes at the cost of efficiency.

One solution to improve the throughput of filters is to incorporate a filter aid, such as diatomaceous earth (DE). DE is made up of micron-scale diatom skeletons that have a unique incompressible and highly porous structure, and there are multiple commercially available types for good manufacturing practice (GMP) manufacturing that are basically inert (Daumke et al. 2019). Although adsorptive depth filters often include DE inside their cartridges, alluvial depth filters leverage DE by adding it directly to the feed stream (also known as body feed filtration) to form a filter cake on top of depth filter media (Scott 2017; Meierrieks et al. 2023). Alluvial filters protect the filter media and dramatically reduce the risk of turbidity breakthrough, as the porous cake serves as the first line of defense to capture cell debris and particulates (Fig. 1b). This additional filtration technique to remove process solids reduces the overall required filter area, as the medium is less prone to clogging. Some instances have shown a 3.5-fold increase in filter capacity when comparing alluvial filtration to centrifugation and depth filtration (Meierrieks et al. 2023). Increased filtration capacity reduces the required disposable filter area, which can lower the consumption of single-use filtration media, although the addition of filter aid contributes additional solids to the waste stream. However, the reduction in disposable filter media can still provide economic and environmental benefits depending on process scale and waste handling strategies. Despite these advantages, filter area remains a limiting factor when scaling alluvial filtration toward production-scale batch volumes, along with the volume of the cake (Ledergerber 2022; Daumke et al. 2019). Alluvial filtration has been described for applications ranging from laboratory scale to production volumes of up to thousands of liters, with increasing scale requiring correspondingly greater filter cake volume and filter area. Once a capsule is filled, it is considered waste and must be replaced with a new capsule, as overfilling alluvial capsules is a considerable concern that could compromise a batch.

To improve the operational efficiency of body feed filtration, multi-cycle cake filtration systems have been developed. This technology utilizes the same advantages of a porous filter cake to increase filter throughput and lifespan, with the added benefit of a cloth regeneration step over multiple cycles (Fig. 1c) (Campbell 1983). Once the cake reaches a maximum volume, a backflush step is performed, cleaning the filter cloth and aiming to restore its original permeability (Morsch et al. 2019). This repeated backflush step can also mitigate the effects of upstream variability, as a denser feed stream can be still processed with the same equipment by simply increasing the number of cycles. Furthermore, the cloth in multi-cycle cake filters is significantly thinner and coarser than depth filter media. Structurally, a thin and coarse cloth not only aids in effective backflush and separation of the filter cake from the cloth, but it also allows for additional benefits: higher flow rates, reduced risk of clogging (Morsch et al. 2021), and no requirement for preflushing.

For clarity, the terminology used to describe filtration processes in this study must be defined. Both alluvial filters and cyclical cake filters involve adding filter aid to the feed to form a filter cake, but they are differentiated throughout this paper in the following way: alluvial filtration refers to body feed filtration performed using depth filter media, and cyclical cake filtration refers to body feed filtration performed using a thin filter cloth, which is designed to be periodically backflushed and reused. Although terminology can vary across the literature, these definitions are used consistently throughout this paper.

While systems such as stainless steel filter presses, leaf filters, and candle filters have implemented cyclical cake filtration techniques for decades (Campbell 1983), a single-use and fully automated cyclical cake filter has only recently been developed, appealing to sterile biopharmaceutical applications. This advancement provides a unique intersection between single-use and multi-cycle technologies, and the cyclical backflush step increases the overall capacity of the filter while maintaining a closed and sterile environment. Because this technology is relatively new to the market, there has yet to be a well-documented study comparing single-use cyclical cake filters to traditional single-use harvesting equipment. This is the content of the current study; cyclical cake filtration is investigated and compared with depth and alluvial filtration with respect to flow rate, filtrate clarity and quality, scalability, and practical implementation.

## Materials and methods

### Cell culture and harvest conditions

#### General cultivation conditions

All experiments were conducted using an immunoglobulin G (IgG)-expressing ExpiCHO-S cell line (Gibco, Waltham, MA, USA). The cultures that were used are summarized in Table 1. Cells were cultivated in Univessel® Glass 10-L bioreactors (Sartorius AG, Göttingen, Germany) controlled by a HyPerforma G3Lab Controller (Thermo Fisher Scientific Inc., Waltham, MA, USA), with 5 to 10 L working volume. All cultivations were inoculated with a viable cell density of 0.3 × 10⁶ cells mL⁻^1^. Across all processes, the temperature was maintained at 37 °C, dissolved oxygen (DO) was kept at 40% via the addition of oxygen through a sparger, and the pH was controlled at ≤ 7.20 by CO₂ addition via the sparger. A constant overlay gassing was applied at 0.1 vvm, and foaming was managed by the addition of a 1:10 diluted Antifoam C solution (Sigma Aldrich Inc., St. Louis, MO, USA).

**Table 1:** ExpiCHO cell cultures used throughout the study. Culture 1 was used for Fig. 4, culture 2 for Fig. 3, culture 3 for Figs. 5, 6, 7, and 8, and culture 4 for Fig. 8 (turbidity data) and Fig. 9

Cultivation no.	Type	Cell density (mio cells/mL)	Viability (%)	Crude turbidity (NTU)	Biomass (%)
1	Batch	9.5	99.2	454	1.0
2	Fed-batch	8.5	75.0	950	3.0
3	Fed-batch	10.7	96.3	870	2.8
4	Perfusion	5.6	69.6	748	2.3

#### Batch and fed-batch cultivations

For inoculum production and the subsequent batch and fed-batch cultivations, Efficient-Pro Medium (Gibco) supplemented with 6 mmol L⁻^1^ L-glutamine and 0.1% anti-clumping agent (ACA, Gibco) was used as basal medium. Efficient-Pro Feed 1 (Gibco) for cultivation 2 and Efficient-Pro Feed 2 (Gibco) for cultivation 3 were used throughout the fed-batch cultivations. For the fed-batch processes, feeding was initiated on day 3 with a daily bolus addition corresponding to 2% of the bioreactor volume. Furthermore, a 450 g L⁻^1^ glucose solution was fed as needed to maintain a minimum glucose concentration of 2 g L⁻^1^.

#### Perfusion cultivation

High-intensity CHO perfusion medium (Gibco) was used for perfusion cultivation. Specifically, a 0.66 × concentration supplemented with 4 mmol L⁻^1^ L-glutamine and 0.1% ACA was used as the basal medium. For the perfusion medium, a 1 × concentration of the medium supplemented with 4 mmol L⁻^1^ L-glutamine and 0.1% ACA was utilized. The perfusion process was initiated on day 3 and increased progressively to maintain a cell-specific perfusion rate (CSPR) of at least 55 pL cell⁻^1^ day⁻^1^ until a maximum perfusion rate of 1.5 volumes of medium per cultivation volume and day (vvd) was achieved. As with the fed-batch cultures, the glucose concentration was kept at or above 2 g L⁻^1^ via the addition of a 450 g L⁻^1^ glucose solution. A cell bleed was initiated once a viable cell volume of 50 mm^3^ mL⁻^1^ was reached.

#### Harvesting procedure

While the cell suspension from the bioreactors was collected at the end of the batch and fed-batch cultures, for the perfusion culture, the cell bleed was collected and diluted with cell-free medium harvested from the perfusion process. All cultures were placed in the fridge after completing the cultivation and filtered on the subsequent day(s).

The first two cultures were used for preliminary tests that were conducted once, while the third culture was used for thorough comparison and scale-up where test conditions were repeated three times (with a few exceptions, as mentioned when relevant). The fourth culture was used for the final scale-up test between different cake filtration systems (repeated three times) and the performance test of cyclical cake filtration across 20 cycles (conducted once).

### Filtration systems and equipment

**Fig. 2:**
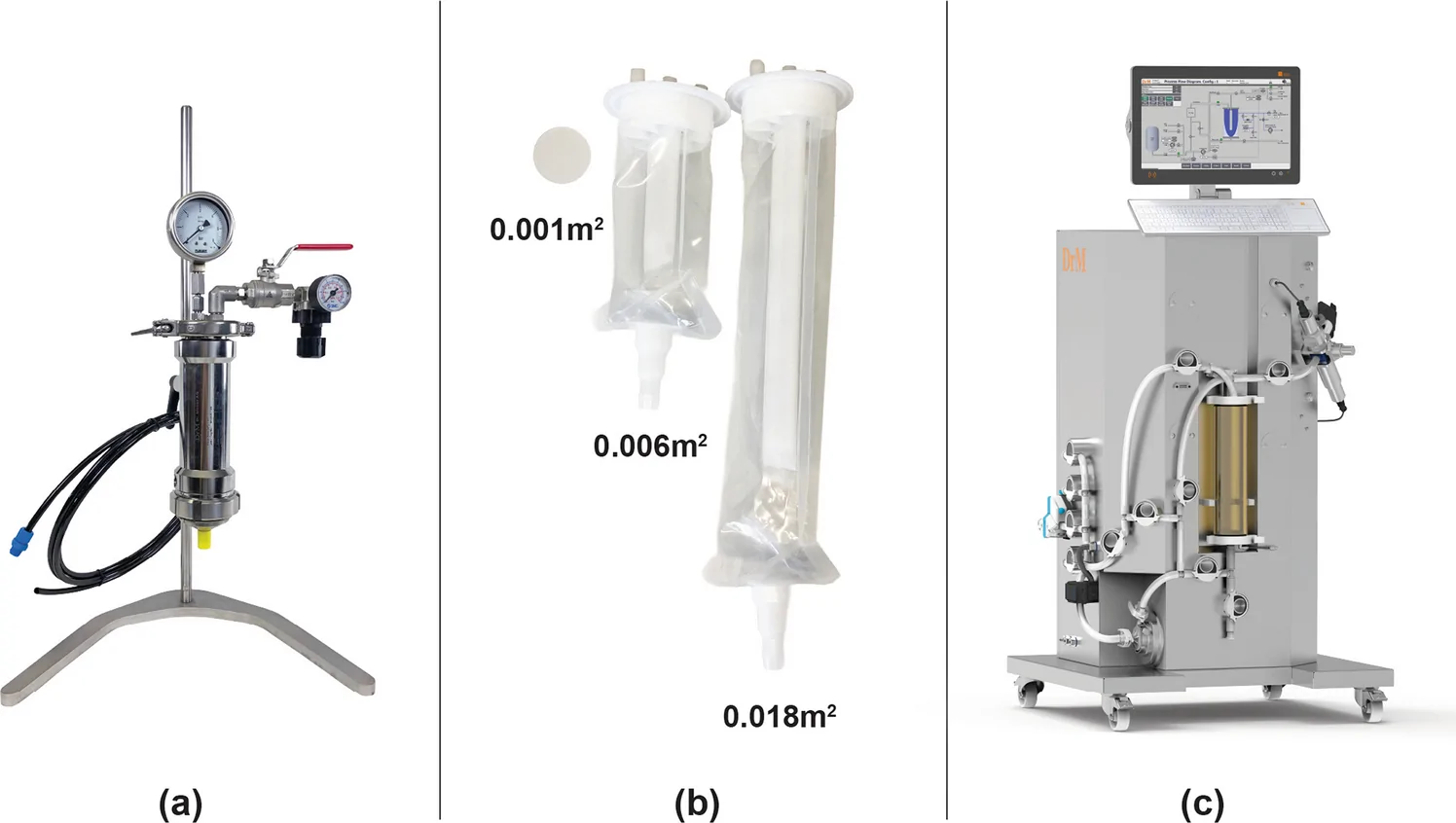
**a** Photo of stainless steel 200 mL Nutsche filter; **b** three scales of the FUNDALOOP® consumable filters: 0.001 m^2^ (left), 0.006 m^2^ (middle), and 0.018 m^2^ (right); **c** fully automatic FUNDALOOP® 50/200 skid

For the cyclical cake filtration, a proprietary polypropylene filter cloth (DrM, Dr. Mueller AG, Männedorf, Switzerland) was used. All experiments with a volume of 200 mL or less were performed in the Nutsche (DrM, Dr. Mueller AG) that is shown in Fig. 2a. The Nutsche consists of a cylindrical body with 200 mL volume underneath which the filter cloth with 0.001-m^2^ cross-sectional area (Fig. 2b) is placed on a porous metal support plate. Pressurized air is applied from the top, and the pressure is set to a constant value via a regulator. The scale-up tests in Figs. 7, 8, and 9 were performed with the FUNDALOOP® skid (DrM, Dr. Mueller AG) shown in Fig. 2c and single-use bags (DrM, Dr. Mueller AG) having 0.006-m^2^ and 0.018-m^2^ cross-sectional filter areas (Fig. 2b). The single-use bags are fitted with a rigid and porous candle element, which is covered with the polypropylene cloth. These bags are securely fitted into the pressure vessel on the FUNDALOOP® skid. Feed is pumped in through the bottom port, and as outside-in filtration commences, filtrate exits through the top port and a cake starts forming on the filter cloth. Depending on the application, it takes between a few seconds and 1–2 min until the cake is fully formed and the filtrate turbidity has reached the desired level. During this time, the filtrate is fed back to the filter vessel in a step that is referred to as “prefiltration.” The cake thickness grows as the filtration continues, and the filtration is terminated once the desired cake volume has been reached. Subsequently, a heel volume filtration step is performed by adding pressurized air into the vessel, which squeezes the bag and filters the remaining liquid through the candle. This is followed by a backflush step with water for injection (WFI) or buffer, which dislodges the cake from the cloth. The cake and backflush liquid are subsequently discharged out of the bag as waste before starting another cycle. A maximum of 50 filtration cycles per filter bag was assumed based on the manufacturer-specified operating limit for the bag film, which reflects the mechanical durability of the film and includes an appropriate safety margin.

For the alluvial filtration, cellulose and diatomaceous earth-based media with retention ranges of 10–30 µm and 0.1–0.3 µm (Filtrox AG, St. Gallen, Switzerland) were used for the primary and secondary clarification steps, respectively. The finer medium was charged with cationization agents. The tested filter area was 0.001 m^2^.

**Fig. 3:**
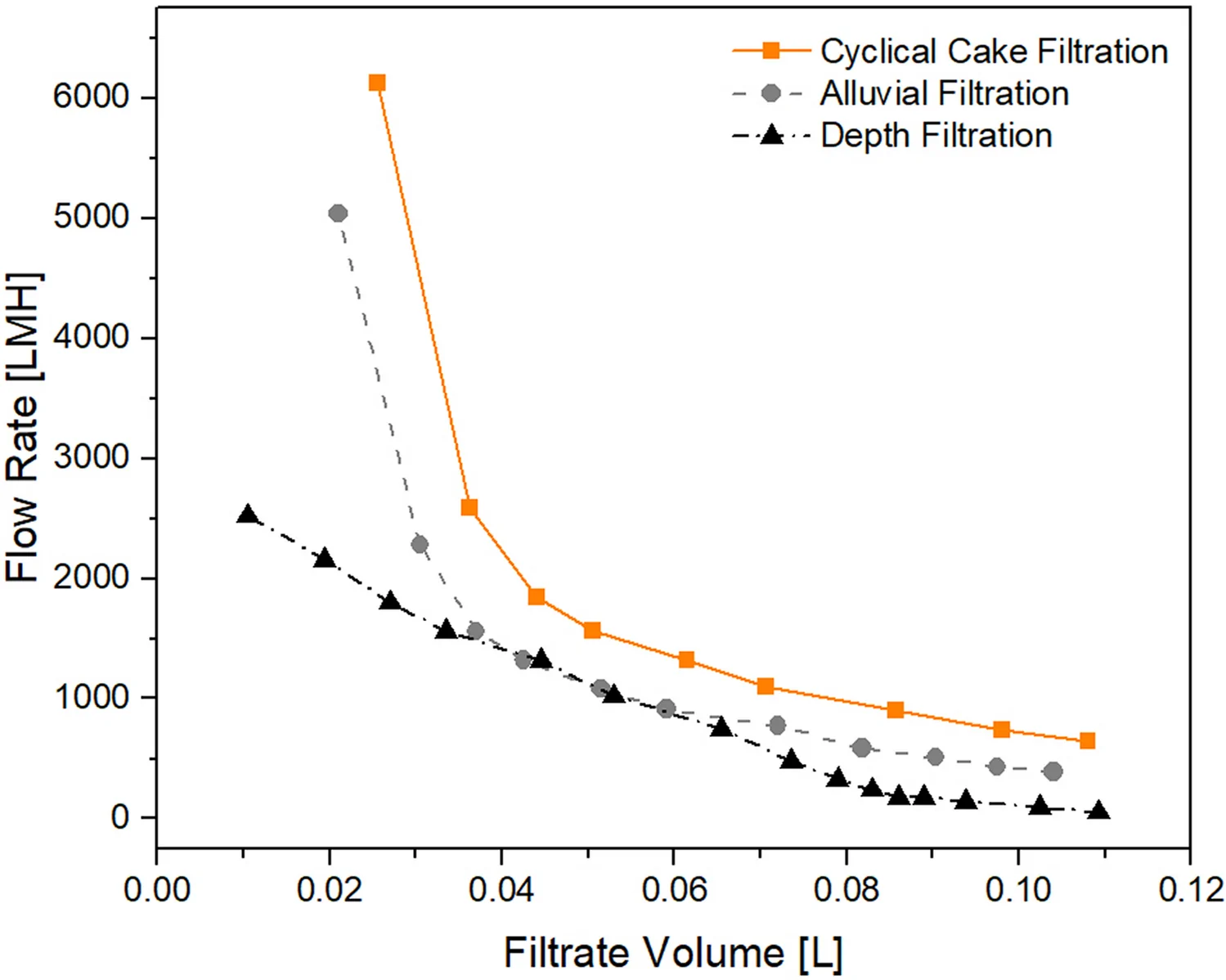
125 mL of culture 2 (Table 1) was filtered using 0.001 m^2^ of depth, alluvial, and cyclical cake filters at a constant pressure of 1.5 bar. 65% DE per biomass was used for the alluvial and cyclical cake filtration, and all tests were performed one time

For the depth filtration, cellulose and diatomaceous earth-based dual layer media with retention ranges of 0.5–5 µm and 0.05–3 µm (3 M, Maplewood, MN, USA) were used for the primary and secondary clarification steps, respectively. The latter again had an anion exchange functionality to adsorb impurities. Lab filter capsules with a filter area of 0.0025 m^2^ were used for the tests, with the exception of the test in Fig. 3, where 0.001 m^2^ was used. A peristaltic pump (Watson Marlow Ltd., Cornwall, UK) with pump head 114DV and 3.2 mm inner diameter tubing was used to establish a constant flow, and a single-use pressure sensor (PendoTECH, Mayfair Technology LLC, Plainsboro, NJ, USA) was used to measure the pressure. The pressure reading was monitored using an LCO-i100 process control unit (Levitronix GmbH, Zürich, Switzerland). To calculate the depth filter sizing in Fig. 7, the P_max method was used (Merck KGaA 2020, see Sect. “Analytical methods”).

The flow rates in all filtration methods were measured indirectly via a scale and taking periodic weight measurements, assuming that the density of the product is identical to that of water. The turbidity of the filtrate was measured in nephelometric turbidity unit (NTU) using a WTW Turb 430 IR/T turbidity meter (Xylem Inc., Washington, DC, USA), and the wet cake thickness in cyclical cake filtration and alluvial filtration was measured using a caliper.

### Diatomaceous earth addition

For the alluvial and cyclical cake filtration, a type of diatomaceous earth (DE) called Celpure® C300 (Imerys S.A., Paris, France) was used. This DE was only added to the feed prior to filtration (body feed filtration); no DE precoat was used for any of the filtration conditions in this study.

For alluvial filtration, 65% (Fig. 3) and 100% (Figs. 5 and 6) per biomass Celpure® C300 were added to the feed, which is within the typical range of 50–100% (van der Meer et al. 2014). This high-purity DE is specifically designed for biopharmaceutical applications and has a nominal permeability of 300 mDarcy. For the cyclical cake filtration, 65–100% per biomass was used. For Fig. 3, 65% was used to have a meaningful comparison with alluvial filtration. For Figs. 5 and 6, 100% was used, while 80% was used for Figs. 7, 8, and 9. Celpure® C300 was again used for all experiments with the exception of one process condition tested in Figs. 5 and 6 where 100% per biomass Celpure® S25E (Imerys S.A., Paris, France) was used, having a nominal permeability of 25 mDarcy. In initial stages of the study, DE was added as a percentage of the wet-cell-weight that was determined by centrifuging 10 mL of cell culture and measuring the remaining cell volume. However, it was discovered throughout the course of these experiments that the calculated biomass was a more consistent measure, and this adaptation ultimately led to the final spread in DE usage between 65 and 100% that was still within the recommended limits.

The biomass was calculated from the total cell count and the average cell diameter, assuming a spherical shape of the cells. For cake washing, phosphate buffered saline (PBS) was used (Roth AG, Arlesheim, Switzerland) that was dissolved in water at a concentration of 9.55 g/L.

### Ion exchange resin screening

During secondary clarification, depth and alluvial filters (which are essentially normal depth filters due to the absence of DE) are typically charged in order to adsorb unwanted impurities such as deoxyribonucleic acid (DNA) and host cell proteins (HCP). In order to study the possibility of removing DNA and HCP with cyclical cake filtration, several ion exchange resins were compared in this study: INDION 810, a macroporous strongly basic type I anion exchange resin; INDION 820, a macroporous strongly basic type II anion exchange resin; INDION 830, a strong base type I anion exchange resin having quaternary ammonium function groups; and INDION 254, a strongly acidic cation exchange resin. All INDION resins were between 0.45 and 0.6 µm and sourced from Indion Exchange Ltd. (Mumbai, India). These ion exchange resins were contrasted with Daicel MabXPure (Daicel Corporation, Tokyo, Japan) that combines anion exchange (AEX) and size exclusion chromatography (SEC) and that has been featured in previous studies (Daumke et al. 2019, Daicel Bioseparations n.d.).

### Filtration experimental design

The first set of tests in Fig. 3 were performed in a Nutsche system (Fig. 2a). In total, 125 mL sample sizes of culture 2 (Table 1) were filtered using 0.001-m^2^ filter area of a depth filter, alluvial filter, and cyclical cake filter at a constant pressure of 1.5 bar, which was chosen to limit cell damage (Minow et al. 2014). Depth filters are not normally run in a pressure-control mode (Sharma et al. 2017); however, the filtration tests were performed this way as it allowed for a direct comparison of the governing filtration mechanisms.

**Fig. 4:**
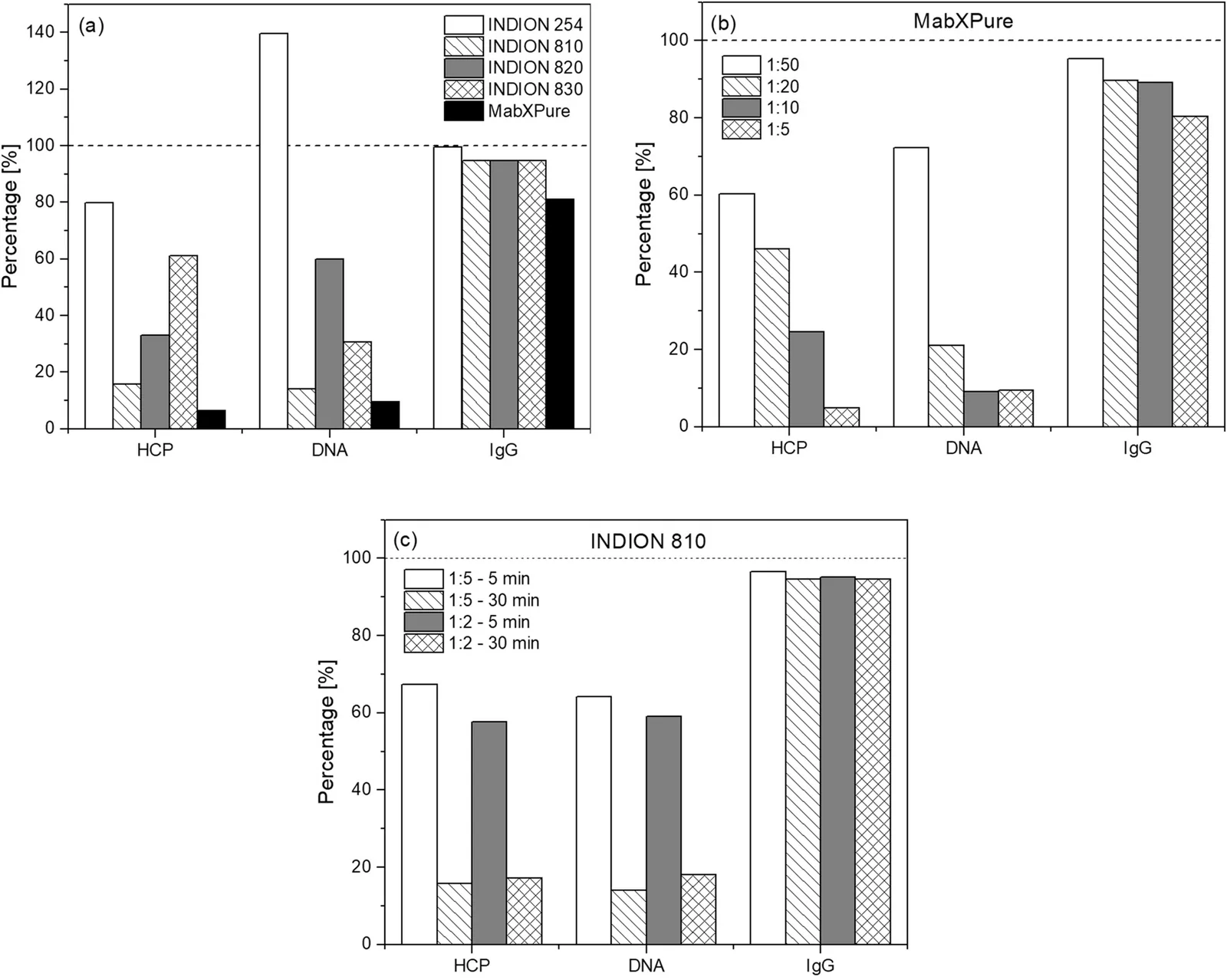
DNA, HCP, and IgG concentrations were compared with addition of various ion exchange resins. 100% refers to the initial concentration of DNA, HCP, and IgG in the feed. For all tests, 160 mL of culture 1 and 100% Celpure® C300 per biomass were filtered in the Nutsche with media from a cyclical cake filter. **a** Five different ion-exchange resins were added to the feed. **b** MabXPure was added in four volumetric ratios (1:50, 1:20, 1:10, 1:5) to the feed. **c** INDION 810 was added in two ratios (1:5 and 1:2) to the weight of DE added, each for two mixing times (5 min and 30 min)

**Fig. 5:**
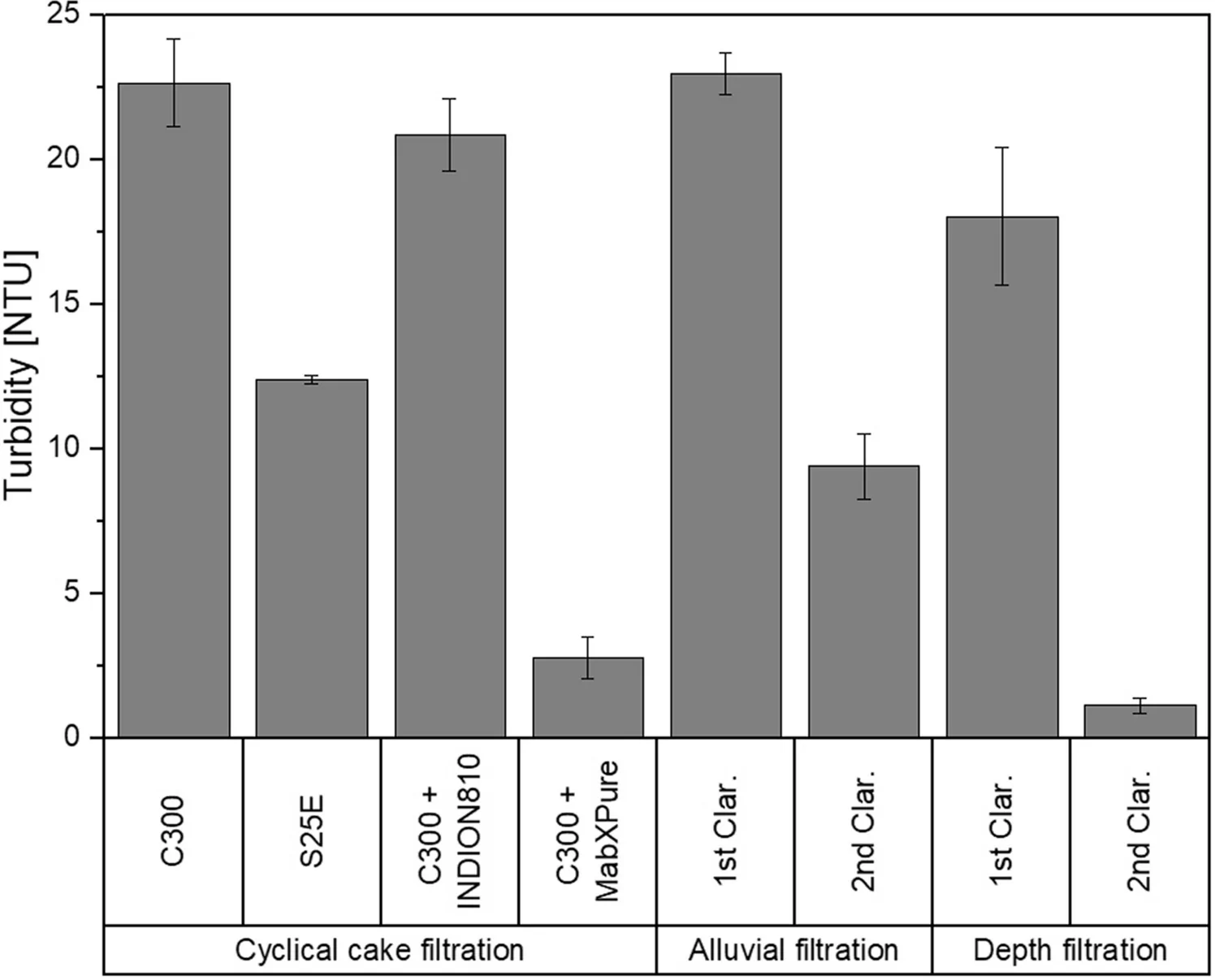
Filtrate NTU was measured after processing culture 3 with cyclical cake filtration in a single stage for four process conditions (always using 100% DE per biomass), and in two stages with alluvial filtration (using 100% DE per biomass) and depth filtration. Depth filters were run with a peristaltic pump at 10 mL/min, or 240 LMH, and alluvial filters were run with a peristaltic pump at 15 mL/min, or 900 LMH, as well as a Nutsche filter. Cyclical depth filters were processed in the Nutsche with a constant pressure of 1.5 bar. The following additions were tested: Celpure® C300, Celpure® S25E with a finer cloth, Celpure® 300 with INDION 810 (1:5 ratio relative to DE), and Celpure® C300 with MabXPure (1:10 ratio relative to feed). Each test was repeated three times, and the error bars represent standard deviations

**Fig. 6:**
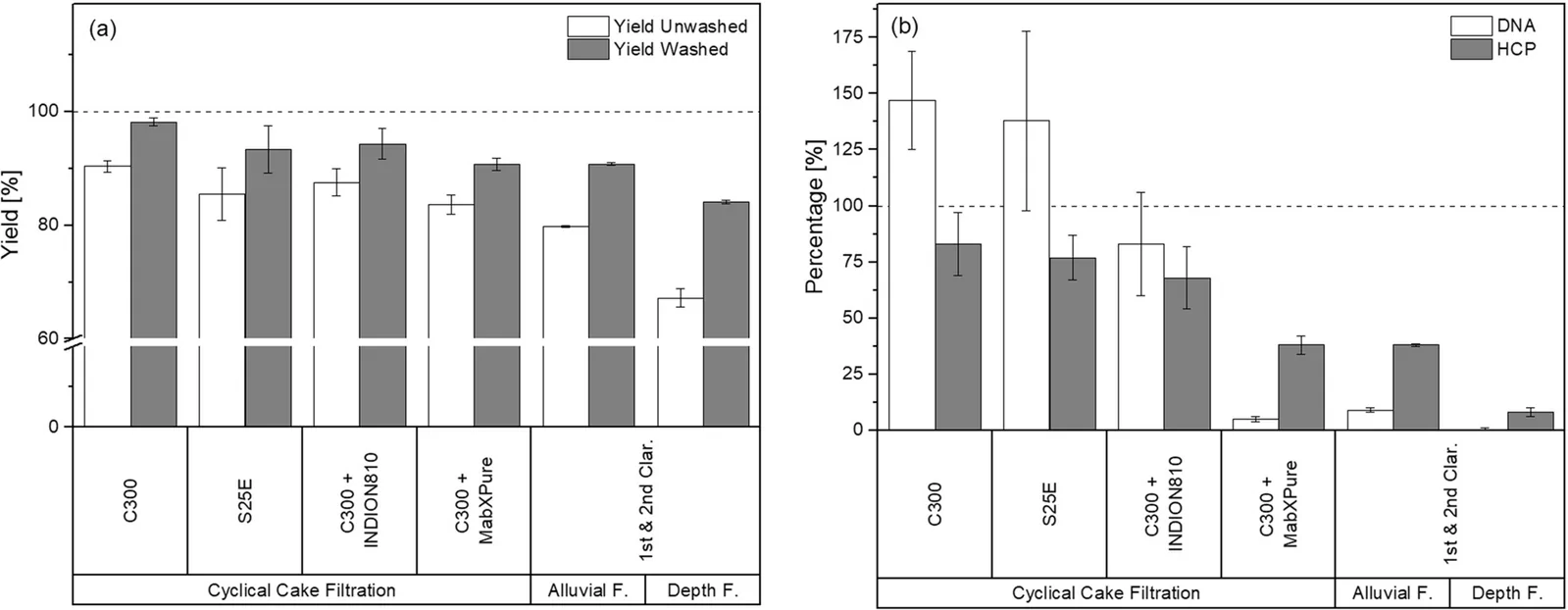
Product yield (based on IgG), DNA, and HCP levels from culture 3 (Table 1) were measured using the same testing conditions as in Fig. 5. **a** IgG levels were measured in the filtrate before and after a PBS wash. For cyclical cake filtration and alluvial filtration, 20% PBS wash per feed volume was used. For depth filtration, 25% PBS wash per feed volume was used. **b** DNA and HCP levels were measured in the filtrate before washing. Each test was repeated three times, and the error bars represent standard deviations

To evaluate the effect of ion-exchange resins on filtrate clarity and quality, five different resins were added to 160 mL samples of culture 1 (Table 1) prior to filtration in the Nutsche with the cyclical cake filter cloth (Figs. 4, 5, 6). All INDION resins were added at ratios 1:2 relative to the weight of DE, and MabXPure was added at a ratio of 1:5 to the feed. In further testing, MabXPure was added in four different volumetric ratios (1:50, 1:20, 1:10, and 1:5) relative to the feed, and INDION 810 was added in two ratios (1:5 and 1:2) to the weight of DE added, each for two mixing times (5 min and 30 min).

**Fig. 7:**
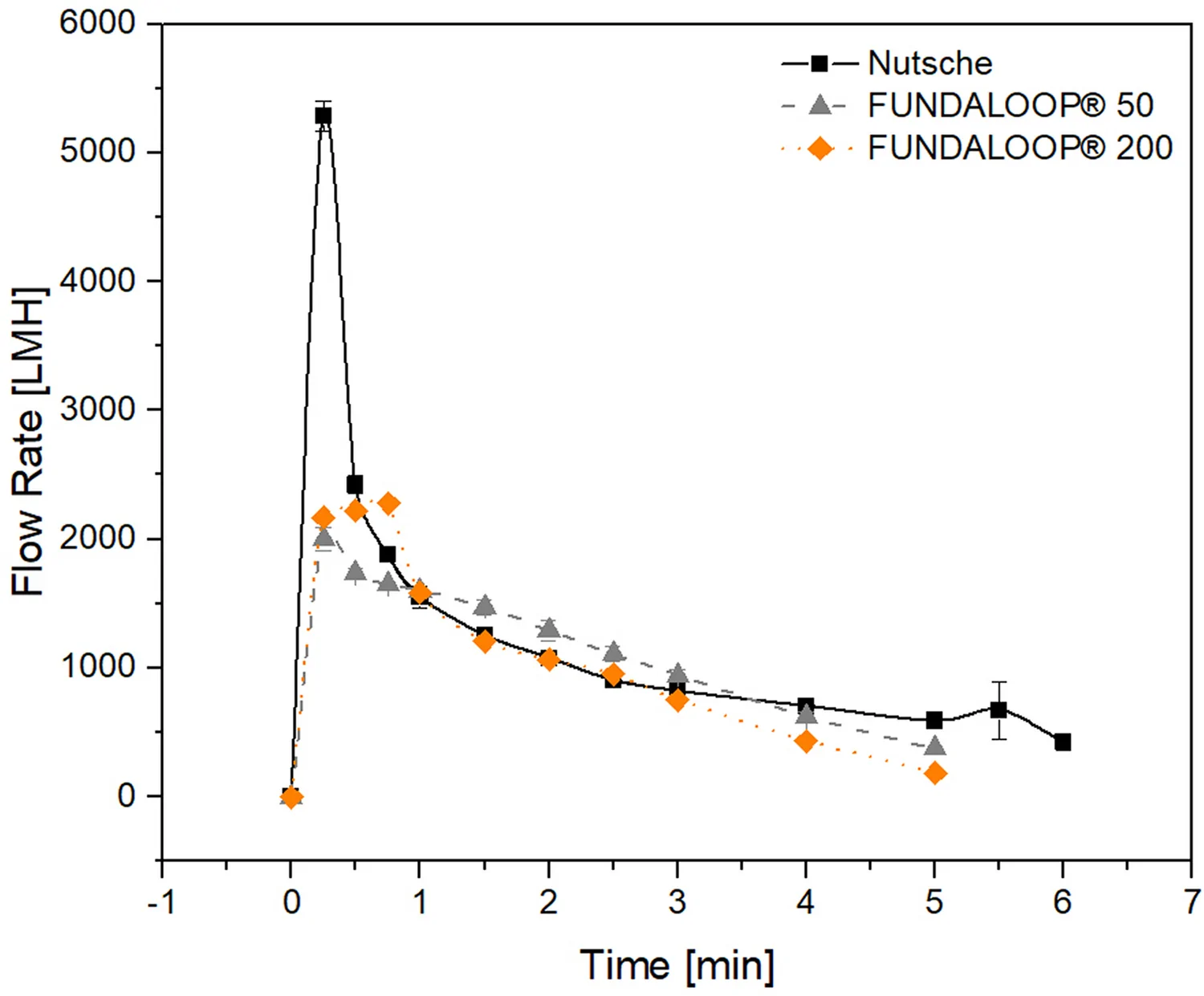
Flow rates in LMH were measured over time in three scales of cyclical cake filtration with culture 3 (Table 1). The filter areas tested were 0.001 m^2^ (Nutsche), 0.006 m^2^ (FUNDALOOP® 50), and 0.018 m^2^ (FUNDALOOP® 200). 80% Celpure® C300 was added per biomass, as well as INDION 810 at a 1:5 ratio relative to DE. The Nutsche and FUNDALOOP® 50 tests were repeated three times, and the error bars represent standard deviations. The FUNDALOOP® 200 test was conducted once

**Fig. 8:**
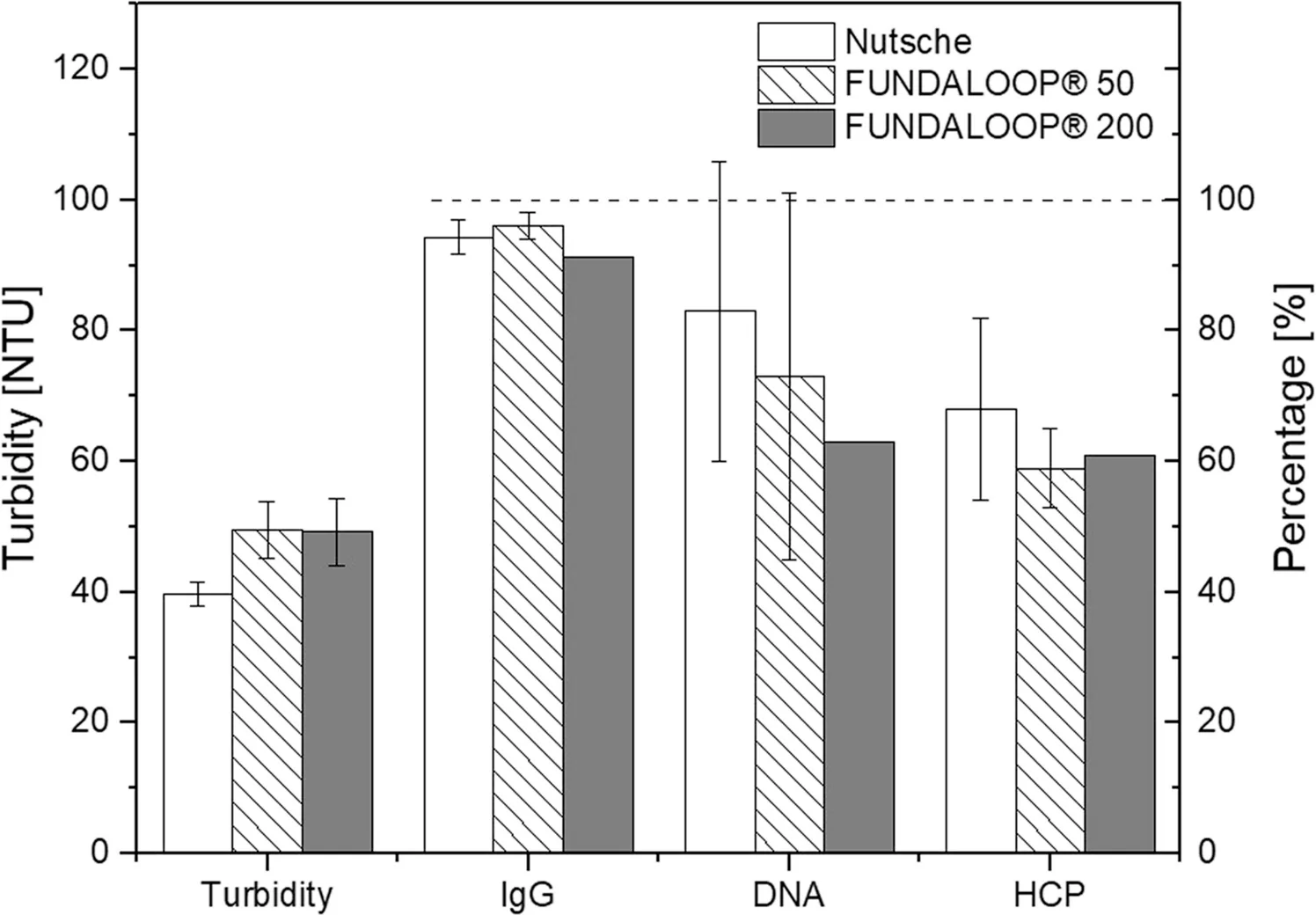
Turbidity, product yield (based on IgG), DNA, and HCP levels were compared over three scales of cyclical cake filtration. Turbidity data was generated using culture 4, while product yield, DNA, and HCP data were generated using culture 3 (Table 1). Turbidity was measured in NTU and the remaining parameters were measured in a relative concentration to the feed material by the same methodology as described in Fig. 4. Celpure® C300 and INDION 810 were added as in Fig. 7. For the FUNDALOOP® 200, IgG, DNA, and HCP tests were conducted once, and turbidity tests were repeated three times. The error bars represent standard deviations

Tests shown in Figs. 5, 6, 7, and 8 were performed with culture 3 (Table 1). For depth filtration, a capacity of 100 mL was determined via *P_max* method outlined in Sect. “Analytical methods,” and this filtration amount was used throughout the tests in Figs. 5 and 6. For alluvial and cyclical cake filtration, 125 mL was filtered based on maximum cake thickness considerations. An exception was made for the condition using Celpure® S25E and a finer filter cloth (DrM, Dr. Mueller AG), where only 75 mL of culture was filtered.

Depth and alluvial filtrations were divided into separate stages for primary and secondary clarification, in which filtrate from the first clarification was collected and measured before being fed into the second filter for further clarification. Both depth filters and alluvial filters were flushed with the prescribed washing liquid prior to filtration (135 mL and 50 mL, respectively). Alluvial filtration was performed using a peristaltic pump for the first test and then was switched to filtration in a Nutsche for the second and third tests due to issues with DE sedimentation in the peristaltic pump tubing. Cyclical cake filters were not pre-flushed as per the operating instructions and were tested using four different process parameters in only one stage.

**Fig. 9:**
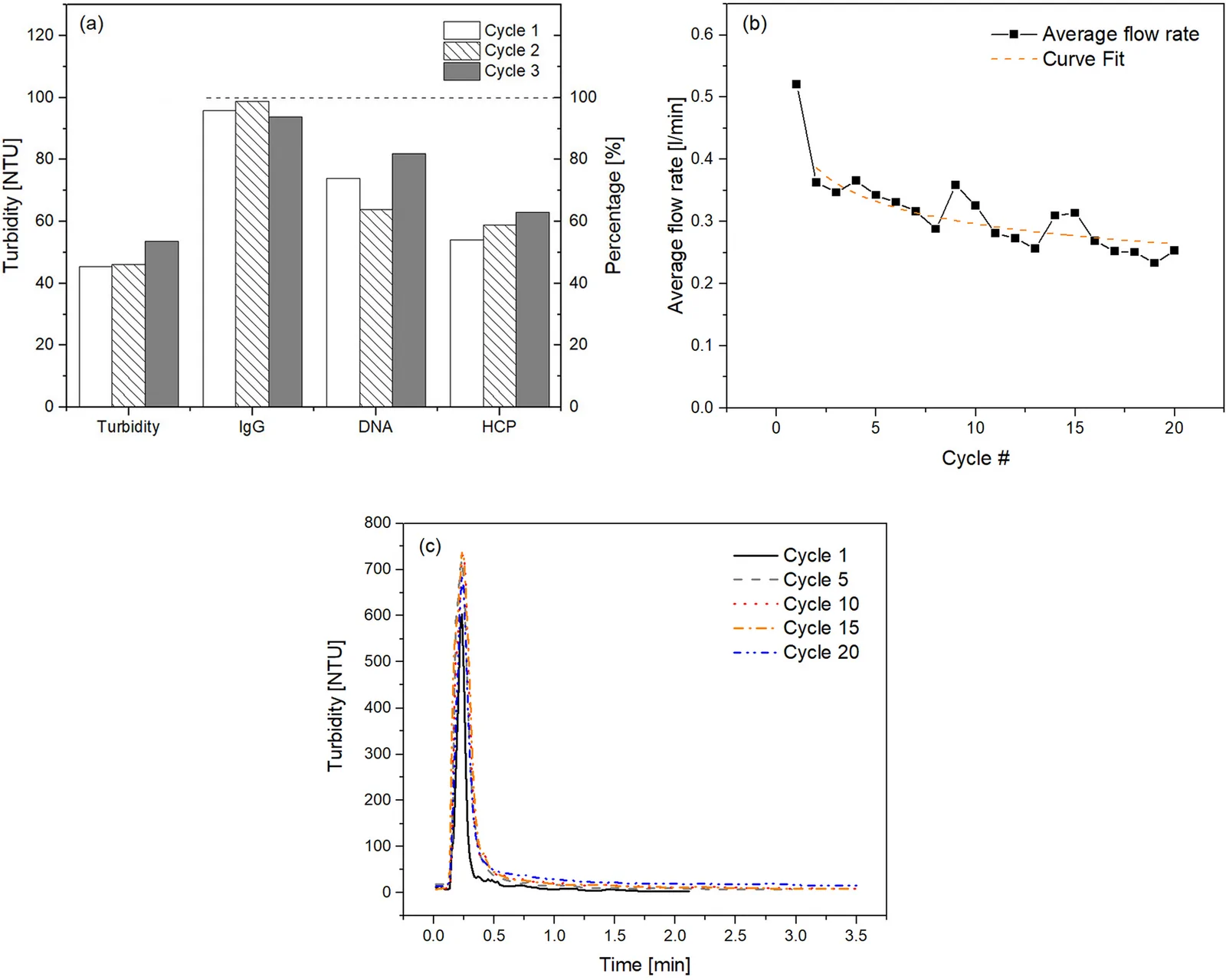
Filtrate across multiple cycles of the FUNDALOOP® 50 was compared. Culture 4 (Table 1) was used and 750 mL was filtered each cycle. **a** Turbidity, IgG, DNA, and HCP were compared over the first three cycles. Over 20 cycles, **b** average flow rate and **c** turbidity were compared. Turbidity was measured in NTU and the remaining parameters were measured in a relative concentration to the feed material by the same methodology as described in Fig. 4

Scale-up tests were performed with culture 3 (Figs. 7 and 8 (igG, HCP, DNA)) and culture 4 (Fig. 8 (turbidity) and Fig. 9) across three sizes of cyclical cake filters: the Nutsche, FUNDALOOP® 50, and FUNDALOOP® 200. The tested volumes at the three scales were 125 mL, 750 mL, and 2250 mL, respectively. Lastly, 20 cycles were run consecutively with the FUNDALOOP® 50 system using culture 4 (Table 1).

### Analytical methods

To evaluate the filter performance, Immunoglobulin G (IgG), HCP, and DNA concentration were measured before and after filtration. Prior to the analysis, the samples were centrifuged at 3000*g* for 5 min to remove any remaining cells. IgG concentration was measured with the IgG Bio test kit (Roche Diagnostics International AG, Rotkreuz, Switzerland) using the Cedex Bio Analyzer (Roche Diagnostics), and product yield was calculated by measuring the concentrations and volumes before and after the filtration. The volume before the filtration was corrected by the volume occupied by cells. For the DNA and HCP measurements, the samples were diluted according to the manufacturer’s recommendation. As the actual concentrations were unknown, three different dilutions were analyzed per sample. HCP concentration was measured using the CHO Host Cell Proteins ELISA Kit (RayBiotech Life Inc., Peachtree Corners, GA, USA). DNA concentration was measured using the Quant-iT Picogreen dsDNA Assay Kit (Life Technologies Corporation, Carlsbad, CA, USA). Both HCP and DNA kits were evaluated using the Varioskan LUX spectrometer (Thermo Fisher Scientific Inc).

To calculate the depth filter sizing in Table 2, the P_max method was used. From the volumetric and pressure data, the resistance was calculated using

1 $$R=\frac{P}{\frac{\Delta V}{A}\Delta t}$$

where *R* is the momentary resistance, *P* the momentary pressure, *∆V* the change in volume over the time interval *∆t*, and *A* the area of the capsule. Additionally, the filter loading was calculated by dividing the total filtered volume by the filter area. A graph was afterwards plotted having the filter loading on the x-axis and the resistance on the y-axis, and a third-order polynomial curve-fit was made. The “actual” resistance *R*_*actual*_ was then evaluated by plugging the capacity (desired batch size divided by the “guessed” filter area *A*_*guess*_) into the polynomial. This value was then compared to the calculated resistance value

2 $${R}_{calc}=\frac{{P}_{limit}}{\frac{{V}_{B}}{{A}_{guess}}{t}_{B}}$$

where *P*_*limit*_ is the maximum allowable pressure, *V*_*B*_ the batch size, and *t*_*B*_ the desired batch time. *A*_*guess*_ was subsequently adjusted until *R*_*calc*_ was equal to *R*_*actual*_.

## Results

Figure 3 shows flow rates of depth, alluvial, and cake filters in L/(m^2^ *h) (LMH) over the course of the filtration volume. Complete blockage of the depth filter occurred after filtration of 125 mL. The highest flow rates were recorded with the cyclical cake filter.

Figure 4a compares the effect of ion exchange resins (detailed in Sect. “Ion exchange resin screening”) on product yield and the removal of impurities. DNA values exceeding 100% may occur because filtration-induced cell damage can lead to lysis and the subsequent release of intracellular DNA, thereby increasing measured DNA concentration. Out of the resins tested, INDION 810 and MabXPure removed the most DNA and HCP and therefore were selected for further investigation in Fig. 4b and c.

Figure 4b compares four different ratios of MabXPure which were added to the feed, showing a direct and significant correlation between the amount of resin added and the removal of DNA and HCP. However, IgG levels were also reduced with a higher ratio of MabXPure. In Fig. 4c, two ratios of INDION 810 were tested, each with two different incubation times. For both ratios of INDION 810, adding the resin 30 min prior to filtration yielded significantly lower DNA and HCP levels than a 5-min incubation time. IgG levels remained quite consistent over the four different testing scenarios.

Figure 5 shows the filtrate turbidity in NTU for the different tested conditions. With the addition of Celpure® C300, the filtrate NTU of cyclical cake filtration was 22.6 - similar to the filtrate NTU after primary clarification in alluvial and depth filtration (23.0 NTU and 18.0 NTU, respectively). The addition of INDION 810 did not have a significant effect on the filtrate NTU; however, both the addition of MabXPure and replacing Celpure® C300 with a finer grade of DE (Celpure® S25E, detailed in Sect. “Diatomaceous earth addition”) resulted in significantly lower filtrate turbidity (2.8 NTU and 12.4 NTU, respectively). The lowest filtrate turbidity of 1.1 NTU was achieved after the second stage of depth filtration.

In Fig. 6a, the same four testing conditions for cyclical cake filtration were compared to alluvial filtration and depth filtration in regard to product yield, both before and after washing with PBS. All tested scenarios showed that a significant amount of product can be recovered with a washing step. Product yield was lowest after depth filtration, at 84.1% after washing, and the highest after cyclical cake filtration, at 90.8–98.2% after washing, depending on the condition tested. The condition which resulted the highest product yield was with cyclical cake filtration and Celpure® C300 alone (98.2%). Figure 6b compares the impurity levels of the filtrate. Depth filters showed the lowest level of both DNA and HCP. Alluvial filtration and cyclical cake filtration with the addition of MabXPure exhibited similar impurity profiles: primary clarification with cyclical cake filters with the addition of MabXPure reduced the concentration of DNA to 5% and HCP to 38%, compared to levels after primary and secondary clarification with alluvial filters which reduced the concentration of DNA to 9% and HCP to 38%. The DNA level after filtration with Celpure® C300 and S25E was higher than 100% because the filtration induces some amount of cell damage that gives rise to an increase in the DNA concentration.

Table 2 proposes an efficiency parameter of different filtration scenarios to process 2000 L of cell harvest based on flow rate data obtained in Fig. 3. A “combined resources needed” value is calculated by incorporating estimated time and filtration area. In the three scenarios, primary clarification is done by cyclical cake filters, alluvial filters, and depth filters, respectively. Secondary filtration for all three scenarios is performed with depth filters. The lower the “combined resources needed” value, the more efficient the system is considered.

**Table 2:** A productivity parameter defined by “combined resources needed” was calculated from estimated filter area and processing time required to clarify 2000 L of CHO culture. A lower value indicates higher efficiency. Cyclical cake, alluvial, and depth filtration were evaluated for primary clarification, with depth filtration proposed for secondary clarification. The calculation assumes that primary clarification feeds directly into secondary clarification in a continuous series, rather than being collected and processed in separate batches

	Cyclical cake filtration	Alluvial filtration	Depth filtration
Time for primary clarification (h)	5.25	1.0	5.0
Time for secondary clarification (h)	5.0	5.0	5.0
Area for primary clarification (m^2^)	0.9	16.8	28.5
Area for secondary clarification (m^2^)	22.4	18.2	14.3
**Combined resources needed**	**122.0**	**175.0**	**213.8**

Depth filtration required the most filter area for primary clarification, as was calculated using a *P_max* calculation (detailed in Sect. “Analytical methods”), assuming a duration of 5-h processing time and a maximum pressure of 1.5 bar (Thakur et al. 2020; Cytiva 2023). These parameters yielded a minimum filtration area of 19 m^2^, and then a 50% safety factor was added as is common practice in the industry (Dryden et al. 2021).

Primary clarification filter area shown for alluvial filtration reflects a maximum cake thickness of 18 mm that was calculated based on the cartridge filter area and allowable cake volume (Filtrox Group n.d.). Based on the Nutsche experiments in Fig. 3, where 125 mL of culture 2 was filtered, it was extrapolated that an 18 mm cake would have been reached at 178.5 mL filtrate volume. Knowing the tested 0.001-m^2^ filter area, it could be calculated that a minimum of 11.2-m^2^ filter area is needed for the filtration of 2000 L. Again, a 50% safety factor was added to obtain 16.8 m^2^. This approximation requires relatively constant solids concentration and limited cake compressibility. From this comparison, it can be seen that less filter area is required for primary clarification with alluvial filters compared to depth filters.

The time for primary clarification with alluvial filters was approximated as 1 h given typical flow rates and estimated resistance in the filters and tubing of a complex system. For the calculation it turned out irrelevant, however, because the bottleneck is the secondary clarification (to be discussed in Sect. “Discussion”) that was assumed to be 5 h since the filtration is fairly similar to the depth filtration scenario.

For cyclical cake filtration, it was determined from experiments run in the FUNDALOOP® 50 that 750 mL can be filtered in one cycle, resulting in an 11 mm cake. This thickness was selected for scale-up calculations as it falls within the manufacturer-recommended maximum cake thickness range (10–12 mm) for reliable discharge performance. Proportionally scaling up the FUNDALOOP® 50 filter area of 0.006 m^2^ to the commercial scale FUNDALOOP® 3000 with a 0.44-m^2^ filter area, it is estimated that two FUNDALOOP® 3000 filter bags with a combined area of 0.88 m^2^ can filter 110 L per cycle, and therefore 18 cycles would be needed to filter 2000 L, assuming constant solids loading and limited cake compressibility.

As per the manufacturer’s specifications and considering flow rates achieved in previous trials, each cycle of 110 L in the cyclical cake filter would take 17.5 min to complete; and thus, processing time to run 18 cycles would be 5.25 h.

Depth filtration is proposed for secondary clarification in all three conditions to maximize DNA and HCP adsorption as shown in Fig. 6. Various references in the literature cite that a 2:1 ratio of primary depth filter area to secondary depth filter area is acceptable (Zustiak et al. 2022; Merck KGaA 2017). Despite having a much cleaner feed stream compared to primary depth filters, the same filtration time was assumed for secondary filtration as the membranes are finer and filtration area is reduced by half.

Because filtrate following primary clarification with alluvial filtration showed approximately 25% higher NTU than filtrate following depth filtration (Fig. 5), 25% greater filter area was considered for secondary clarification after alluvial filtration. A similar comparative result was observed with cyclical cake filtration in the Nutsche (Fig. 5). Given that large-scale cyclical cake filtration systems produce NTU values approximately 25% higher than those achieved in the Nutsche (see Fig. 8), the required filter area for secondary clarification following cyclical cake filtration was estimated to be an additional 25% greater than that required following alluvial filtration.

The “combined resources needed” (Table 2) parameter was ultimately obtained by multiplying the combined primary and secondary filter area times whichever filtration (primary or secondary clarification) time that took longest, under the assumption that primary and secondary clarification are performed in series as discussed in Sect. “Discussion”.

Figure 7 compares flow rates between three scales for cyclical cake filtration: the Nutsche, FUNDALOOP® 50, and FUNDALOOP® 200. The tested volumes at the three scales were 125 mL, 750 mL, and 2250 mL, respectively. With the exception of a brief initial surge in flow recorded in the Nutsche, the flow rates exhibited similar patterns and values between scales.

Filtrate quality parameters were compared between the same three scales for cyclical cake filtration in Fig. 8. Product yield was consistent and similar across all scales, ranging from 91.2% to 96.1%. DNA and HCP were less consistent than yield, but within range of each other, especially considering the larger standard deviations associated with the measurement. Turbidity was around 25% lower in the Nutsche than the FUNDALOOP® 50 or 200.

In Fig. 9, average flow rate and filtrate clarity were compared over 20 cycles in the FUNDALOOP® 50, with a more in-depth screening for product yield and impurity levels performed on filtrate from the first three cycles. Figure 9a shows there was no clear trend in product yield or DNA concentration over the first three cycles, although HCP concentration did increase with each cycle, from 54% concentration in the first cycle to 63% in the third. Average flow rates over 20 cycles did decrease logarithmically, and the curve fit line appeared to stabilize and plateau over time (Fig. 9b). Filtrate turbidity values exhibited a similar trend across all 20 cycles (Fig. 9c), showing an initial surge of NTU during the prefiltration in approximately the first 25 s that corresponds to the time required for a cake to form, and then stabilizing at a reduced turbidity.

## Discussion

A critical element of efficiency in bioproduction involves how quickly a batch can be processed. Faster flow rates during cell harvesting and clarification are one way to decrease processing time and increase efficiency. As shown in Fig. 3, flow rates with depth filter media started off slower and continued to slow down in a nearly linear fashion until the flow rate was close to 0, where it stayed until it eventually became completely clogged. This can be explained by the mechanism of capture; cells and debris are retained by the thick depth filter medium itself, which leads to rapid clogging (Datta and Redner 1998).

In contrast, for the same filtrate volume, no clogging occurred in alluvial filtration, as the cells and debris are primarily held back by the filter cake, which protects the filter media and preserves its capacity (Sørensen et al. 1997). Unlike in depth filtration, the alluvial filtration capacity is limited by the maximum cake thickness — a physical constraint of the filter — rather than clogging of the porous structure (Daumke et al. 2019).

In cyclical cake filtration, the flow rates were even a bit higher compared to alluvial filtration (Fig. 3). However, this can likely be attributed to the initial filtration conditions. In cyclical cake filtration, the filter cloth is thin (around 0.5 mm) and coarse with minimal initial resistance, whereas in alluvial filtration, the filter cloth is much thicker (2–4 mm) with much higher initial resistance. As the filtration progresses and the cake grows, the filter cake resistance becomes increasingly dominant over the filter cloth resistance; hence, the two flow rates start declining along similar trends. Because the Nutsche experiments were conducted under controlled pressure conditions, normalization for initial permeability was not performed.

The maximum capacity per cycle in cyclical cake filtration could be even higher than in alluvial filtration; however, the capacity is usually limited by the maximum recommended cake thickness from the manufacturer — typically between 10 and 12 mm. Nonetheless, the overall capacity and average flow rate are increased with the allowance of 50 cycles to be performed using one single-use consumable. Overall, Fig. 3 demonstrates that cyclical cake filtration significantly decreases the process risk compared to depth filtration during the primary clarification step. The flow rate of depth filtration dropped to 0 after filtering 125 mL, signifying that the filter was clogged. In a production setting, if a depth filter clogs before a harvesting batch is complete, this either requires early termination of the filtration, leading to product loss, or an intervention to replace the filter, introducing contamination risk and additional downtime. The incorporation of filter aid and repetitive backflushing of the cyclical cake filter keeps flow rates high and eliminates the risk of clogging — one of the key downfalls of depth filters that often leads to a partial loss of product titers. Additionally, there is no risk of overfilling filters, as is a concern in alluvial systems, because the filter is always regenerated before it reaches its cake volume holding capacity. Variability in the production or feed streams can therefore be effectively handled without adding more filter area, and instead just increasing the number of cycles to process a batch. This approach improves process efficiency while also reducing overall process risk by eliminating clogging and overfilling concerns.

While this study shows that depth filters struggled to achieve high throughput and would require an exorbitant amount of filter area at scale, they are known to capture a large portion of impurities with electrostatic interactions by incorporation of a charged media (Charlton et al. 1999). However, this advantage could be rivaled in alternative filtration methods by the addition of ion exchange resins to the feed. Five different resins are evaluated in Fig. 4a for impurity removal after cyclical cake filtration. It can be assumed that INDION 254 did not work well, or even at all, as the DNA and HCP values were similar to those seen in Fig. 6b, where impurity levels were measured after cyclical cake filtration with no resin addition and only Celpure® C300.

Considering the results from Fig. 4a, the two most promising resin options for impurity reduction in cyclical cake filtration are investigated further in Fig. 4b and c: MabXPure and INDION 810. The addition of MabXPure at a 1:5 ratio of resin to feed reduced the DNA and HCP levels the most, albeit at an expense to the product yield. Reducing the ratio of MabXPure to 1:10 increased the product yield to approximately 90% while also removing a majority of the impurities (Fig. 4b). Furthermore, INDION 810 resulted in a higher product yield of approximately 95%, and still removed the majority of DNA and HCP even with a low ratio, as long as the resin was incubated for 30 min prior to filtration. IgG levels were unaffected by INDION 810 ratio or incubation time.

While a reduced impurity profile helps to reduce the burden of further downstream steps (Nejatishahidein and Zydney 2021; Parau et al 2022), other characteristics of high filtrate quality include filtrate clarity and product yield. These parameters were compared between cyclical cake filtration, alluvial filtration, and depth filtration in Figs. 5 and 6. It is important to note that alluvial filtration and depth filtration were each run in a two-stage filter train, standardly referred to as primary and secondary clarification. The data shown from cyclical cake filtration is obtained from only primary filtration.

For a standard filtration where 80% Celpure® C300 per biomass was used, the turbidity of cyclical cake filtration roughly matched the turbidity achieved after the first clarification with alluvial and depth filters. With the addition of MabXPure in a 1:10 ratio of resin to feed and 80% Celpure® C300 per biomass, the turbidity was significantly reduced to less than 5 NTU and rivaled the filtrate clarity of depth filtration after primary and secondary clarification (Fig. 5). The possibility to achieve similar clarity levels in one step versus two could have significant implications on cost, time, and complexity of cell harvesting in a bioprocess. Additionally, it can be expected that if a second filtration run was performed with cyclical cake filters, turbidity would be further reduced.

Another cyclical cake filtration condition shown in Fig. 5 that reduced filtrate NTU was using a finer DE grade (Celpure® S25E) and a finer cloth. Compared to Celpure® C300, the smaller size and lower permeability of Celpure® S25E are hypothesized to aid in capturing smaller micron-sized particles. This DE substitution yielded a filtrate NTU of approximately 12 in a single step, similar to the level which was reached after the second stage of alluvial clarification. This trade-off reflects a well-documented phenomenon in filtration systems, where tighter filter media typically yield improved filtrate clarity at the expense of increased pressure buildup and reduced flow rate (Chandler and Zydney 2005). These parameters are interdependent and therefore must be carefully balanced when optimizing filtration performance.

In addition to reducing the filtrate NTU, MabXPure also significantly improved the impurity profile of the filtrate (Fig. 6b). The levels of DNA and HCP in the filtrate from cyclical cake filtration with the addition of MabXPure were nearly identical to that of alluvial filtration. Although depth filtration still resulted in the lowest impurity profile, the addition of MabXPure provides an alternative option to reduce DNA and HCP without depth filtration. It is prudent to note that data shown in Fig. 6b was measured pre-washing with PBS, and future studies must be done to measure the impurity levels post washing to understand if this would increase DNA or HCP levels. It is possible that the addition of ion exchange resins in the cake would tightly bond impurities and therefore retain them well even during washing, but this needs to be investigated further.

It also needs to be noted that due to the aforementioned challenges with DE sedimentation in the peristaltic pump tubing (Sect. “Filtration experimental design”), the second and third alluvial filtrations were carried out in a Nutsche. These tests were therefore subjected to higher flow rates than are suggested by the manufacturer due to operational limitations of the Nutsche. It is probable that if alluvial filtration had been conducted with slower flow rates, longer contact time with the filter media would have resulted in more effective capture of DNA and HCP, similar to the levels shown with depth filtration. This would be best confirmed in a future study.

Although the surface charge on depth filter media can aid in impurity removal, it is non-specific and can therefore capture any molecule that it has an electrostatic interaction with. This can include the product of interest, in turn resulting in a lower product yield (Khanal et al. 2018). This is exemplified in Fig. 6a, with depth filters having the lowest product yield compared to alluvial and cake filtration. Product can also be retained by the filter cake and cloth in cyclical cake filtration and alluvial filtration, but the effect of this is less pronounced. The highest product yield was observed after cyclical cake filtration with the addition of Celpure® C300 without ion exchange resin. The finer DE grade and additional resins resulted in a slightly lower product yield, albeit higher than depth filtration and alluvial filtration. It must be noted again that these results are from only primary clarification with cyclical cake filters, and if secondary clarification is required afterwards, product yield would likely be further reduced, the extent of which depending on the secondary clarification method selected. Nevertheless, preliminary measurements did indicate a roughly 5% increase in yield prior to PBS washing between cyclical cake filtration (89.6%—shown in Fig. 6a) and the primary clarification of depth filtration (84.7%—not shown), but further studies will be required to investigate this topic in greater detail.

When considering all results of Figs. 5 and 6, cyclical cake filtration with the addition of Celpure® C300 and MabXPure was able to achieve similar results as two-stage depth or alluvial filters in terms of impurity removal and filtrate clarity, albeit at a higher product yield than depth filters.

These results were obtained using IgG as the model product. Because different molecules exhibit distinct isoelectric points (pI) and adsorptive properties, product yield may vary substantially depending on the process conditions (Westoby et al. 2011). Therefore, thorough optimization is necessary to determine which filter aids and additives, if any, are most suitable for each product and process.

Furthermore, although these findings demonstrate that the addition of MabXPure can reduce impurity levels in the filtrate while achieving a high product yield, the cost of resin additives could offset the benefits. Harvest conditions and requirements should be evaluated individually to determine if resin addition is a valuable and economic option.

It can be argued that filtrate quality is the most critical measure of filtration performance, but practical implementation will often depend on a proper scale-up evaluation. In Table 2, data was extrapolated from small scale trials (Fig. 3) to estimate required filtration area and processing time to filter a 2000-L batch, commonly used as a commercial scale production volume when using single-use bioprocessing equipment. These values were then used to calculate a “combined resources needed” value, which serves as an efficiency parameter for each filtration method, where the system requiring fewer combined resources to process a batch is considered more efficient.

Although primary clarification of each scenario was proposed to be run with cyclical cake, alluvial, and depth filters, respectively, secondary clarification for each scenario was recorded to be processed with depth filters, for both consistency and considering the importance of impurity removal in most commercial settings. Furthermore, to maximize efficiency and reduce the risk of contamination, it was assumed that primary and secondary clarification were run directly in series as opposed to processed in separate batches (Schmidt et al. 2017). This meant that the most time-intensive step between primary and secondary clarification is considered the rate-limiting step of the harvesting process and therefore used in the “combined resources needed” calculation. This brings to light an important reality in the implementation of harvesting equipment: reducing the duration of a single step does not always reduce the time of the overall process. In this case, although alluvial filters can process the primary feed stream relatively quickly, the overall harvesting time is governed by the secondary clarification step performed with depth filters.

The second variable accounted for in the proposed productivity parameter is filter area. Alluvial filtration significantly reduces primary filter area requirement compared to depth filters, as the filter cake extends the life of the media. Further supporting this, Minow et al. (2014) reported that scaling up depth filters to commercial levels often times does not make financial sense due to low filtration capacities per filter area. However, both depth filters and alluvial filters are typically oversized and installed with a safety factor to protect against upstream variabilities in solid content or in case of early clogging (Laska et al. 2005; Thakur et al. 2020; Dryden et al. 2021). In alluvial filters, this safety factor is often intended to avoid overfilling of a capsule, whereas in depth filters it primarily prevents filter blockage.

Cyclical cake filtration leverages the benefit of a protective cake layer in alluvial filtration even further through the inclusion of a regenerative backflush step, substantially reducing the required surface area for primary clarification. Moreover, no additional safety factor was applied in the calculation for primary clarification, as each cyclical cake filter is designed by the manufacturer to withstand up to 50 cycles. The 18 cycles estimated to process 2000 L in Table 2 therefore correspond to a 177% margin relative to the maximum allowable number of cycles. Based on these assumptions, using cyclical cake filtration for primary clarification could allow for up to 96% and 94% filter area savings compared to depth and alluvial filtration, respectively, although actual reductions will depend on process conditions.

Required filtration area directly correlates to operational cost, potential for extractables and leachables to compromise a batch, and quantity of plastics and single-use materials that are discarded after each batch. Furthermore, both capital equipment requirement and cleanroom space also increase with higher consumable usage. Implementation of cyclical cake filtration as a harvest method may offer economic and environmental benefits, allowing for a lean and efficient bioprocess step.

Material and operational cost, waste, and cleanroom space were not included in the “combined resources needed” parameter due to lack of all available data, even though they are ultimately necessary to complete the picture. Still, the comparison in Table 2 clearly suggests that using cyclical cake filtration as a primary clarification method can lead to a significant improvement in the overall harvest efficiency.

It needs to be mentioned that this study did not conduct any optimization on the depth and alluvial filtrations and simply compared models that are standard in the industry for both. In recent years, those competing technologies have been continuously developed further by manufacturers, resulting in products on the market that achieve better results in terms of capacity, yield, and removal of impurities. However, those filters still have the same drawbacks (can only be used once until clogging, are not regenerable, and require a large filter area), and it is expected that general trends in the data remain the same.

Scalability between individual cyclical cake filtration systems is shown in Figs. 7 and 8, detailing flow rates and filtrate quality. Between the Nutsche system and FUNDALOOP® 200, there is an 18-fold increase in the filter area. In Fig. 7, flow rates across all three systems behaved similarly, apart from an initial high flow rate in the Nutsche as the operational mechanism differed, and 1.5 bar was achieved instantly. In the FUNDALOOP® systems, pressure built up more slowly to account for bag filling and formation of the cake, resulting in a lower initial flow rate. After pressure was established, flow patterns between the three systems were similar.

The quality profile of filtrate was comparable between the three scales as well (Fig. 8). While slight variations in impurity levels were observed, the large standard deviation values indicate a high degree of experimental variability and the need for further investigation. Product yield, however, was relatively unaffected by scale. The turbidity was around 25% lower in the Nutsche system, which could be attributed to the absence of turbulence inside the filter vessel. In the FUNDALOOP® system, the feed was introduced from the bottom of the vessel, generating upward flow that promoted mixing and maintained a homogeneous suspension during filtration. In contrast, filtration in the Nutsche occurred in the direction of gravity and may have allowed a thin layer of filter aid to effectively form a pseudo-precoat at the start of filtration, due to the quick settling tendency of diatomaceous earth. The observed variation of filtrate clarity was likely a function of this mechanistic difference in vessel hydrodynamics as opposed to differences in the filtration area of each scale. Within the FUNDALOOP® 50 and 200 sizes, the turbidity values were stable and consistent.

A larger system, the FUNDALOOP® 3000, is also commercially available and exhibits approximately a 24-fold increase in filtration area compared to the FUNDALOOP® 200. While tests with the FUNDALOOP® 3000 were outside the scope of this study, it shares the same operational and hydrodynamic characteristics as the smaller size FUNDALOOP® systems, and the presented data likely supports the scalability of this system as well.

As the FUNDALOOP® systems are designed to be used in a cyclical manner, performance characteristics were compared over multiple cycles. Figure 9a provides a comparison of filtrate quality measures during the first three cycles. There was not a noticeable trend seen in product yield or DNA concentration, but turbidity and HCP concentration did increase from cycle 1 to cycle 3. However, a further look into turbidity at regular intervals over 20 cycles (Fig. 9c) suggests that the general trend of turbidity across the length of each cycle is similar, and most notably, the amount of time that it took during the prefiltration for the turbidity to drastically reduce and plateau was consistently around 30 s, with the exception of the first cycle, which resulted in a reduced stable turbidity at around 18 s. After cycle 1, this data suggests that a dependable prefiltration time can be expected, leading to more predictable operating conditions.

Although a reliable prefiltration time contributes to process consistency, the average flow rate over the course of filtration has the greatest influence on a consistent cycle time. While the backflush step serves as a unique advantage to regenerate the filter cloth and restore permeability, complete recovery is idealistic and not achievable, and it is expected that some fine particles inevitably become embedded in the filter cloth with repeated use. Abdelrasoul et al. (2013) describe backwashable versus non-backwashable fouling, noting that backwashable fouling is reversible and can be removed by reversing the direction of flow across the filter, whereas non-backwashable fouling may require a chemical cleaning step to remediate. The imperfect nature of the backflush step was reflected in the logarithmic decline in the average flow rate over 20 cycles (Fig. 9b), suggesting that some non-backwashable fouling may have occurred. The curve fit line suggests that the system approaches a steady-state flow rate at approximately 0.25 L/min for the condition tested in the FUNDALOOP 50®. From an operational perspective, these results indicate that while some reduction in throughput should be expected over time, the rate of this decline becomes less pronounced, allowing for more predictable cycle times as the filtration continues.

## Conclusion

This study demonstrated that cyclical cake filtration is an efficient and scalable alternative to depth and alluvial filtration for CHO harvest clarification, providing higher flow rates, high product yield, reduced process risk, and significantly lower filtration area requirements. The use of filter aids protects the filter cloth and mitigates the clogging limitations commonly associated with depth filters. The thin filter medium and regenerative cycling improve throughput, while the incorporation of ion exchange resins such as MabXPure or INDION 810 enables substantial removal of DNA and HCP, achieving filtrate clarity and impurity levels approaching those of multi-stage depth or alluvial filtration in a single-use unit operation. Additionally, scale-up studies demonstrated comparable filtration performance across three different cyclical cake filter scales. Collectively, these results highlight cyclical cake filtration as a robust clarification technology capable of reducing risk and consumable requirements while enhancing midstream bioprocess efficiency.

## Data Availability

All data supporting the findings of this study are included in the article. Additional datasets generated during the current study are available from the corresponding author upon reasonable request.

## Abbreviations

ACA: Anti-clumping agent
AEX: Anion exchange
CHO: Chinese hamster ovary
CSPR: Cell-specific perfusion rate
DE: Diatomaceous earth
DNA: Deoxyribonucleic acid
DO: Dissolved oxygen
GMP: Good manufacturing practice
HCP: Host cell protein
IgG: Immunoglobulin G
LMH: L/(m^2^ *h)
NTU: Nephelometric turbidity unit
PBS: Phosphate buffered saline
SEC: Size exclusion chromatography
vvd: Volumes of medium per cultivation volume per day
vvm: Volumes of gas per volume of liquid per minute
WFI: Water for injection
